# Retropharyngeal effusion secondary to calcific tendinitis of the longus colli muscle: A case report and brief review of the literature

**DOI:** 10.1016/j.radcr.2024.12.063

**Published:** 2025-01-10

**Authors:** Musharaf Khan, Subtain Ali, Dhairya A Lakhani

**Affiliations:** Department of Neuroradiology, Rockefeller Neuroscience Institute, West Virginia University, Morgantown, WV, USA

**Keywords:** Longus colli tendonitis, Retropharyngeal effusion

## Abstract

Calcification or ossification of the longus colli tendon is a rare and often underdiagnosed cause of acute neck pain, typically seen in middle-aged adults, especially women. This condition results from the deposition of calcium hydroxyapatite crystals in the tendon, causing an inflammatory or granulomatous response. Common symptoms include neck pain, painful swallowing, and fever. CT imaging is the preferred diagnostic tool, showing retropharyngeal and prevertebral effusions with minimal or no peripheral enhancement, often accompanied by calcifications in the superior fibers of the longus colli tendon. This condition can be mistaken for a retropharyngeal abscess, which requires more invasive management, such as drainage. In contrast, calcific tendinitis responds well to conservative treatment, including nonsteroidal anti-inflammatory drugs (NSAIDs). Here, we report a case of retropharyngeal effusion secondary to calcific tendinitis of the longus colli muscle, highlighting the importance of distinguishing it from other conditions, such as retropharyngeal abscess, to avoid unnecessary invasive treatments.

## Background

The calcification or ossification of the longus colli tendon is an uncommon and often underdiagnosed cause of acute neck pain. It is an inflammatory or granulomatous response to the deposition of calcium hydroxyapatite crystals in the tendons of the longus colli muscle. This condition typically affects middle-aged adults between the ages of 30 and 60, with a higher prevalence in women. Patients commonly present with neck pain, painful swallowing, and fever [[1], [2], [3], [4]].

CT of the neck is the diagnostic modality of choice, as it reveals retropharyngeal and prevertebral space effusions with minimal or no peripheral enhancement. It almost always shows calcification of the longus colli tendon, particularly in the superior fibers at the C1-C2 level. On CT imaging, retropharyngeal effusion can be differentiated from a retropharyngeal abscess due to the absence of peripheral enhancement. Other conditions that can present with effusion include prevertebral hematomas, often related to trauma, or bleeding associated with tumors. The presence of longus colli tendon calcification or ossification, lack of peripheral enhancement, absence of osseous destruction, and no soft tissue component all support the diagnosis of calcific tendinitis, which is often misdiagnosed as an abscess in clinical practice [[5], [6], [7], [8], [9], [10]].

Management of calcific tendinitis is typically conservative, with nonsteroidal anti-inflammatory drugs (NSAIDs) and resolution usually occurring within a few weeks. In contrast, a retropharyngeal abscess requires more aggressive management, including drainage and debridement [2,[7], [8], [9],^11^].

Here, we present a case of retropharyngeal effusion secondary to calcific tendinitis of the longus colli muscle, which can mimic a retropharyngeal abscess—an entity that requires more invasive treatment.

## Case report

A 44-year-old male with no significant past medical history presented to the emergency department with a one-week history of neck pain and odynophagia. The patient was afebrile, and his vital signs were within normal limits. On examination, there was mild erythema of the pharyngeal wall without exudate, and the tonsils were not enlarged. Initial laboratory workup was unremarkable, except for a mildly elevated C-reactive protein level of 26 mg/dl.

**Fig. 1:**
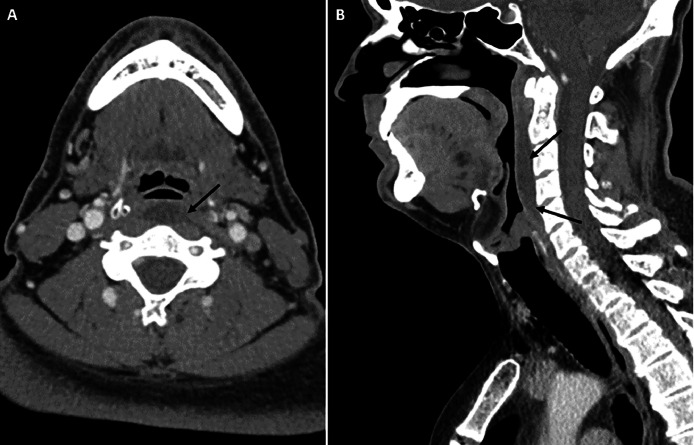
Contrast-enhanced CT of the neck in the soft tissue window, showing axial (A) and sagittal (B) images, demonstrating a large retropharyngeal effusion without peripheral enhancement (black arrows).

**Fig. 2:**
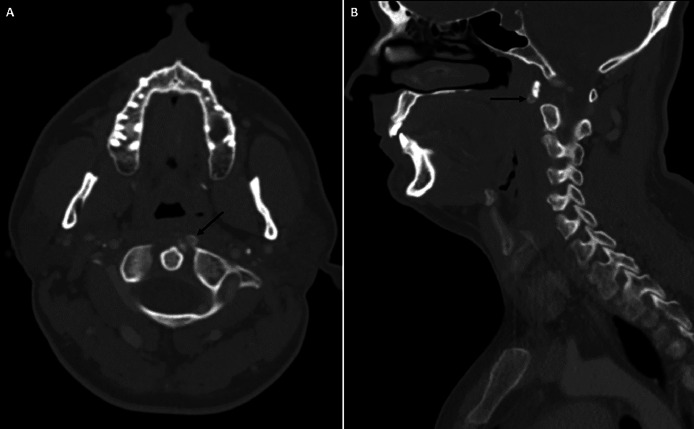
Contrast-enhanced CT of the neck in the bone window, showing axial (A) and sagittal (B) images, highlighting calcification within the superior fibers of the left longus colli muscle tendon at the C1/C2 level (black arrows).

Further evaluation with CT of the neck revealed a moderate-sized retropharyngeal effusion and edema of the adjacent prevertebral soft tissues (Fig. 1). Amorphous calcifications were also noted in the superior fibers of the left longus colli tendon at the C1-C2 level (Fig. 2). There was no enhancement around the effusion, which was most suggestive of longus colli calcific tendinitis with a reactive retropharyngeal effusion. No cervical or retropharyngeal adenopathy or osseous destruction was observed, making other differential diagnoses, such as retropharyngeal abscess, tumor, or post-traumatic hematoma, less likely.

**Fig. 3:**
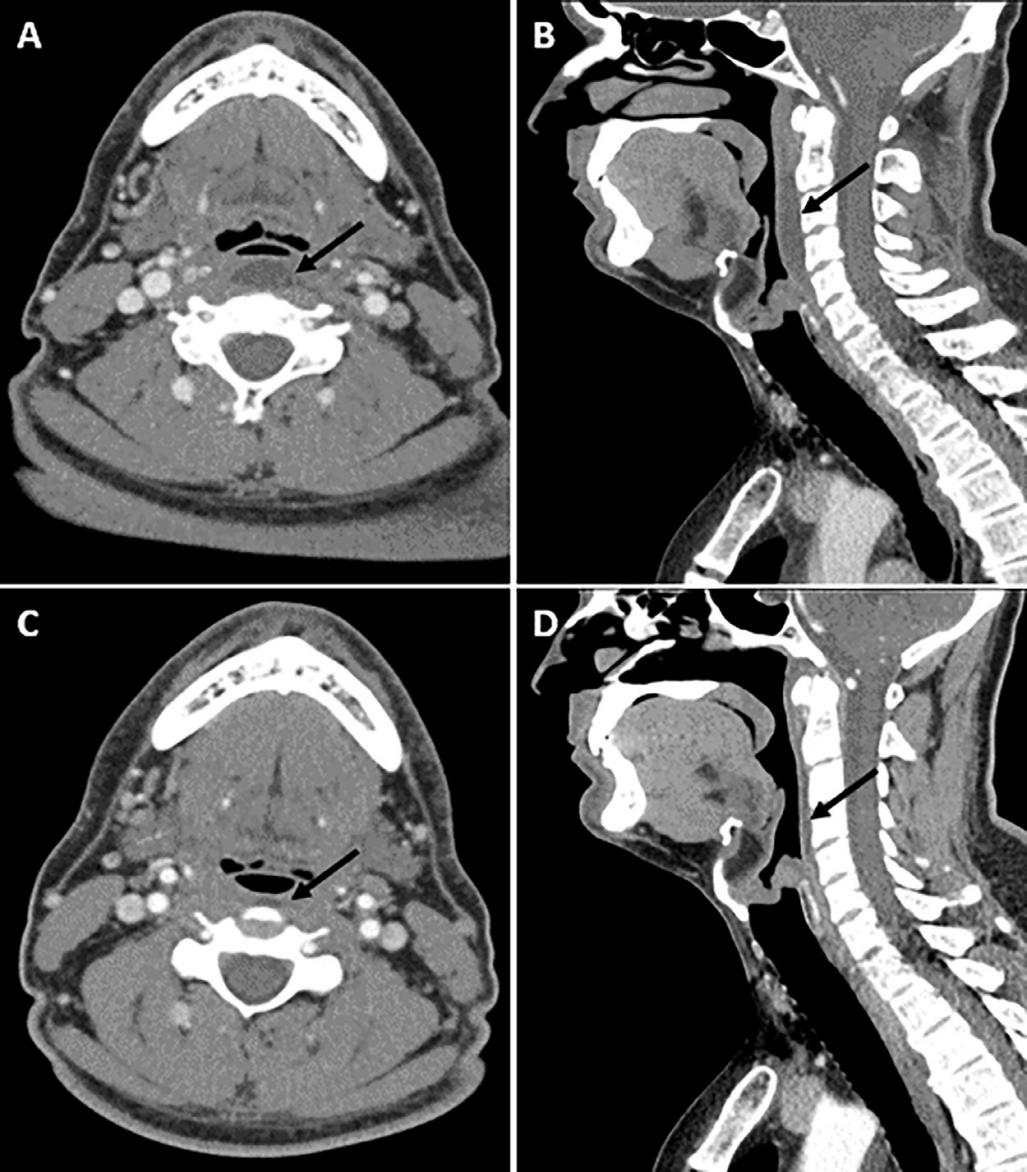
Initial CT of the neck, showing axial (A) and sagittal (B) reformatted images, and repeat CT of the neck, showing axial (C) and sagittal (D) images performed 5 days later at the same level, demonstrating complete resolution of the retropharyngeal effusion (black arrows).

The patient was treated with nonsteroidal anti-inflammatory drugs and oral empiric antibiotics. A repeat CT of the soft tissues of the neck with contrast, performed five days later, showed near-complete resolution of the retropharyngeal effusion (Fig. 3), along with improvement in symptoms.

This case highlights a retropharyngeal effusion secondary to calcific tendinitis of the longus colli muscle, which can mimic a retropharyngeal abscess—a condition that requires more aggressive invasive treatment.

## Discussion

The retropharyngeal space extends from the skull base to the mediastinum and typically contains fat and lymph nodes. Fluid accumulation in this space can result from either noninfectious retropharyngeal edema or retropharyngeal infections, such as suppurative retropharyngeal nodes or abscesses. CT imaging with contrast is the modality of choice for accurate and timely diagnosis. Notably, retropharyngeal effusion can be differentiated from a retropharyngeal abscess on CT by the presence of calcification or ossification in the longus colli muscle, as well as the lack of significant peripheral enhancement around the effusion [1,[3], [4], [5],^7^,^9^].

Calcific tendinitis occurs due to the deposition of calcium hydroxyapatite crystals in the longus colli muscle, likely resulting from repeated trauma, tissue necrosis, or ischemia. The rupture of these crystals triggers an inflammatory response, which can lead to the formation of reactive fluid in the retropharyngeal space, surrounding the muscle. Although this condition is rare, it is often misinterpreted or confused with other pathologies. With the increasing availability of CT imaging in emergency departments, more cases are likely to be diagnosed in the future. This condition predominantly affects individuals aged 30 to 60 years and is more common in women. Patients typically present with acute cervical pain, often accompanied by reflex muscle contraction, reduced cervical range of motion, and varying degrees of painful swallowing. On physical examination, findings are usually subtle, with mild erythema of the pharyngeal wall being the most common sign. Laboratory results are often normal, though some patients may present with mildly elevated C-reactive protein levels. Cervical spine radiographs typically reveal amorphous calcifications at the superior insertion of the longus colli tendon at the C1-C2 level, along with prevertebral soft tissue swelling extending from C1 to C4 (or, less commonly, up to C6), which may displace the airway anteriorly. CT imaging demonstrates retropharyngeal fluid gently expanding in all directions, with the absence of wall enhancement—contrast that would be present in a retropharyngeal abscess. Additionally, calcifications in the longus colli muscle tendons are characteristic [6,7,10,12,13].

Calcific tendinitis generally responds well to conservative treatment, including nonsteroidal anti-inflammatory drugs (NSAIDs), cervical immobilization, and a soft or crushed diet to manage associated dysphagia. In more severe cases, intravenous corticosteroid therapy may lead to rapid clinical improvement. Typically, the condition resolves within 1-2 weeks. In contrast, a retropharyngeal abscess—often a mistaken diagnosis in these cases—requires more invasive treatment, including drainage and debridement [[2], [3], [4], [5],[7], [8], [9]].

## Conclusion

In conclusion, the presence of calcification in the superior fibers of the longus colli muscle, coupled with the absence of peripheral enhancement, should favor the diagnosis of retropharyngeal effusion secondary to calcific tendinitis of the longus colli muscle, rather than a retropharyngeal abscess.

## Patient consent

Informed consent was obtained by MK granting permission to publish this case report.
